# Pre-Existing Humoral Immunological Memory Is Retained in Patients with Multiple Sclerosis Receiving Cladribine Therapy

**DOI:** 10.3390/biomedicines9111584

**Published:** 2021-10-31

**Authors:** Tobias Moser, Ciara O’Sullivan, Christian Puttinger, Julia Feige, Georg Pilz, Elisabeth Haschke-Becher, Janne Cadamuro, Hannes Oberkofler, Wolfgang Hitzl, Andrea Harrer, Jörg Kraus, Eugen Trinka, Peter Wipfler

**Affiliations:** 1 Department of Neurology, Christian Doppler University Hospital, Paracelsus Medical University and Center for Cognitive Neuroscience, 5020 Salzburg, Austriachristian.puttinger@stud.pmu.ac.at (C.P.); j.feige@salk.at (J.F.); georg.pilz@salk.at (G.P.); a.harrer@salk.at (A.H.); e.trinka@salk.at (E.T.); p.wipfler@salk.at (P.W.); 2 Department of Laboratory Medicine, Paracelsus Medical University, 5020 Salzburg, Austria; e.haschke-becher@salk.at (E.H.-B.); j.cadamuro@salk.at (J.C.); h.oberkofler@salk.at (H.O.); joerg.Kraus@outlook.com (J.K.); 3 Research Management (RM): Team Biostatistics and Publikation of Clincial Studies, Paracelsus Medical University Salzburg, 5020 Salzburg, Austria; wolfgang.hitzl@pmu.ac.at; 4 Department of Ophthalmology and Optometry Salzburg, Paracelsus Medical University Salzburg, 5020 Salzburg, Austria; 5 Research Program Experimental Ophthalmology and Glaucoma Research, Paracelsus Medical University, 5020 Salzburg, Austria; 6 Department of Dermatology and Allergology, Paracelsus Medical University Salzburg, 5020 Salzburg, Austria; 7 Department of Neurology, Medical Faculty, Heinrich-Heine-University, 40225 Düsseldorf, Germany; 8 Neuroscience Institute, Christian Doppler University Hospital, Paracelsus Medical University and Center for Cognitive Neuroscience, 5020 Salzburg, Austria

**Keywords:** vaccination, lymphodepletion, immune reconstitution, titers, immunization, antibody levels, infections

## Abstract

Cladribine (CLAD) is a lymphodepleting agent approved for active relapsing multiple sclerosis (MS). The impact of CLAD on the adaptive humoral immune system has not sufficiently been studied. This study aimed to assess the influence of CLAD treatment on specific antibody titers to common pathogens. We included 18 MS patients treated with CLAD. Serum IgG antibody levels to measles, mumps, rubella, hepatitis B and varicella zoster virus (VZV), as well as diphtheria and tetanus toxins, were measured prior to the initiation of treatment and at 12 and 24 months after first CLAD administration. Moreover, specimens were longitudinally analyzed regarding absolute blood concentrations of IgG and main lymphocyte subsets. No reduction in antibody levels against measles, mumps, rubella, VZV, hepatitis B, diphtheria toxin and tetanus toxin associated with CLAD treatment was observed. Loss of seroprotection occurred in <1%. We found no significant impact of CLAD on absolute serum IgG levels. Absolute lymphocyte counts were significantly reduced at the end of each treatment year (*p* < 0.00001 and *p* < 0.000001). This study suggests that CLAD does not interfere with the pre-existing humoral immunologic memory in terms of pathogen-specific antibody titers.

## 1. Introduction

Cladribine (CLAD) tablets are an approved treatment for highly active multiple sclerosis (MS) in Europe, and relapsing forms of MS (relapsing MS, active secondary progressive MS) in the United States. It is administered in two short treatment courses approximately one year apart. CLAD constitutes a member of immune reconstitution therapies (IRTs), which induce a profound but transient lymphopenia with the rationale of resetting the detrimental, proinflammatory dysregulations in MS [1]. CLAD primarily depletes memory B and T helper 17 (Th 17) cells, which are considered to be the main culprits of MS [2,3], while regulatory subsets and immune cells important for pathogen control are relatively spared [4,5,6]. In fact, CLAD is highly efficient in reducing MS disease activity, yet infectious adverse events have not been a major concern [7,8,9]. Although adaptive and innate immune cells are major contributors to host defense, strong evidence exists that the humoral immunity—in particular, neutralizing antibodies—prevent serious infection [10,11]. In fact, antibody titers are frequently assessed to measure the efficacy of immunizations and to estimate the susceptibility to vaccine-preventable diseases [12]. The main source of antibodies are terminally differentiated B cells called plasma cells, underscoring the close interplay between the cellular and molecular components of the immune system. While cellular dynamics associated to CLAD treatment have been extensively studied, the impact of IRTs on the humoral axis of the immune system remains largely elusive [4,5,6]. One report assessed intrathecal antibody production in 29 treatment-naïve MS patients, which was clearly impaired by subcutaneous CLAD [13]. This observation naturally raises the question of whether protective antibody titers in the blood are similarly affected by the mode of action of CLAD. The aim of this study was to evaluate whether CLAD induces changes in the pre-existing humoral immunologic memory, which, in turn, would have implications on vaccine strategies—we did not account for new humoral immunologic memory during CLAD. Therefore, we monitored serum antibody levels to common pathogens before and during the first two years of CLAD therapy. Moreover, we longitudinally studied blood immunoglobulin G (IgG) concentrations and main lymphocyte subsets.

## 2. Materials and Methods

### 2.1. Participants

In this prospective observational study, we recruited 18 patients with active relapsing MS who had consented to treatment with oral CLAD at the outpatient MS clinic of the Christian Doppler University Hospital of Salzburg between 2017 and 2020. CLAD was administered in the approved dose and according to the manufacturer’s recommendations. We collected demographic data including age, sex, duration of MS and baseline EDSS score. Patients were clinically examined at 3-month intervals and interviewed for opportunistic infections.

### 2.2. Ethics

The study was approved by the local ethics committee (Landesethikkommission Salzburg 415-E/1612/11-2018 on 17 October 2018). All patients gave written informed consent.

### 2.3. Blood Analysis

Peripheral venous blood was collected into 3 mL EDTA tubes (Greiner Bio-One, Kremsmünster, Austria) and 10 mL serum tubes (Becton Dickinson, Franklin Lakes, NJ, USA) immediately prior to treatment start as well as at 12 and 24 months after the initial CLAD administration. The serum tube was centrifuged for 10 min with 3000× *g* at room temperature. Subsequently, serum aliquots were stored at −80 °C for later batch processing. EDTA whole blood was analyzed immediately. All analyses were performed by standard routine laboratory methods at the local Department of Laboratory Medicine, certified according to the ISO-9001 standard and working according to ISO-15189 standards.

ELISA (enzyme-linked immunosorbent assay) or CMIA (chemiluminescent microparticle immunoassay) tests were used to detect specific IgG antibody levels to measles, mumps, rubella, varicella zoster virus (VZV), hepatitis B, diphtheria and tetanus toxins (instruments and assay details are provided in Appendix A Table A1). Total immunoglobin concentrations (IgG) were measured by kinetic nephelometry using the Siemens BNII device (Siemens Healthineers, Erlangen, Germany) and according reagents (N Antiserum gegen IgG, Siemens Healthineers).

EDTA whole blood was used to assess absolute lymphocyte counts on the Sysmex XN system (Sysmex, Kōbe, Prefecture Hyōgo, Japan) and for subtyping T helper cells (Th, CD4+), cytotoxic T cells (cT, CD8+) and B cells (CD19+) by flow cytometry (BD FACSLyric, Becton Dickinson).

### 2.4. Statistical Analysis

Data were checked for consistency and normality by using normal probability plots and Kolmogorov–Smirnov tests. Data were log-10 transformed and geometric means were computed when appropriate. The delta method for computation of 95% CI of geometric means was used. Mixed models with time as the repeated factor were used to analyze continuously distributed variables. LSD tests were used for paired comparisons. Whisker plots were used to illustrate results. All reported tests were two-sided, and *p*-values < 0.05 were considered statistically significant. All statistical analyses in this report were performed by use of NCSS (NCSS 10, NCSS, LLC. Kaysville, UT, USA) and STATISTICA 14 (Hill, T. and Lewicki, P. Statistics: Methods and Applications. StatSoft, Tulsa, OK, USA).

## 3. Results

### 3.1. Patient Characteristics

We enrolled 18 MS patients assigned to CLAD treatment in this prospective 24-month observational study. All patients reached the first follow-up at 12 months (T1) and 12 (67%) reached the second follow-up at 24 months (T2). The reasons why six patients were not included at the second follow-up were: in four patients, redosing of CLAD was delayed because of the COVID-19 pandemic; one patient was switched after the first treatment year due to ongoing disease activity; and one patient was lost to follow-up.

Of the 18 patients, 14 were female (78%). The mean age was 36.7 years (SD: ±8.76 years) with a median EDSS of 1.75 (IQR 1.0–3.5) at treatment initiation. Six patients had been treated with other MS agents within six months from CLAD start (dimethyl fumarate and glatiramer acetate *n* = 2, respectively; daclizumab and fingolimod *n* = 1, respectively). Demographics of the CLAD cohort are shown in Table 1.

### 3.2. CLAD Has No Effect on Pathogen-Specific Blood Antibody Levels

**Figure 1:**
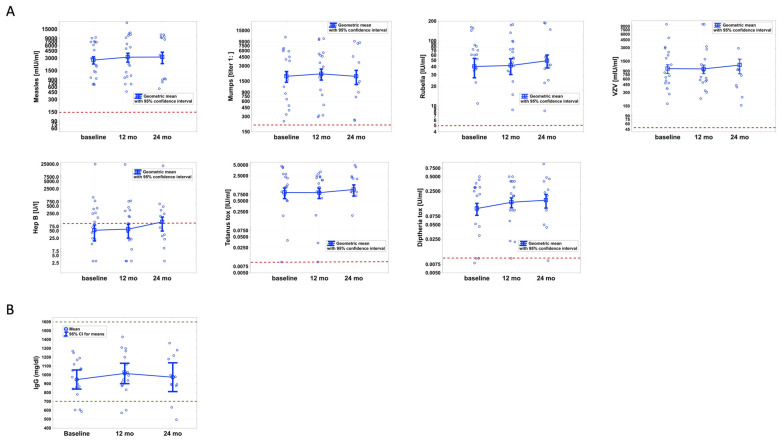
Specific IgG antibodies to 7 pathogens were not altered by cladribine (CLAD) treatment at 12 and 24 months (mo) (**A**). Absolute IgG blood levels remained stable despite immune reconstitution by CLAD (**B**). No significant reductions were found. CLAD tablets were administered at baseline and months 1, 12 and 13 according to the manufacturer’s recommendations. Red dotted lines indicate the cut-offs for antibody titers. Black dotted lines represent the normal range.

The main goal of this work was to study the impact of CLAD on pre-existing IgG antibody levels against common clinically important pathogens including measles, mumps, rubella, varicella zoster virus (VZV), hepatitis B as well as diphtheria and tetanus toxins (Figure 1A). We found no significant alterations over time for any pathogen-specific IgG level. We next investigated whether individual patients lost seroprotection to specific pathogens during the 24-month follow-up. At baseline, the respective cut-off criteria (Table A1) for seroprotection were met in 100% for measles, mumps, VZV, and tetanus toxin and 94% for diphtheria toxin and rubella, and 50% were seroprotected against hepatitis B at T0. Throughout the study period, seroconversion was observed in two patients. The first had antibody titers against hepatitis B of just above cut-off (>100 U/L) at baseline (116 U/L) and at T1 (101 U/L), which decreased to 68 U/L at 24 months from CLAD start. In the second, baseline titers against diphtheria toxin were 0.008 (cut-off: ≥0.01 U/mL) and increased to 0.01 U/mL at T1, before falling to just below the cut-off at T2 (0.009 U/mL). In summary, seroprotection was lost in 0.8%.

### 3.3. Blood IgG Synthesis Is Not Altered during CLAD Therapy

The absolute serum IgG levels at baseline were within the normal range (700–1600 mg/dL) in 15/18 (83%; all but #1, #5 and #16) of all patients. A hypogammaglobulinemia was found in 2/18 patients (11%, #5 and #16) at 12 months and in 2/12 patients (17%, #5 and #7) at 24 months from CLAD start. Overall, we observed no significant reductions in absolute blood IgG levels associated with CLAD treatment (Figure 1B).

### 3.4. T Cells Are Significantly Reduced after 12 and 24 Months

**Figure 2:**
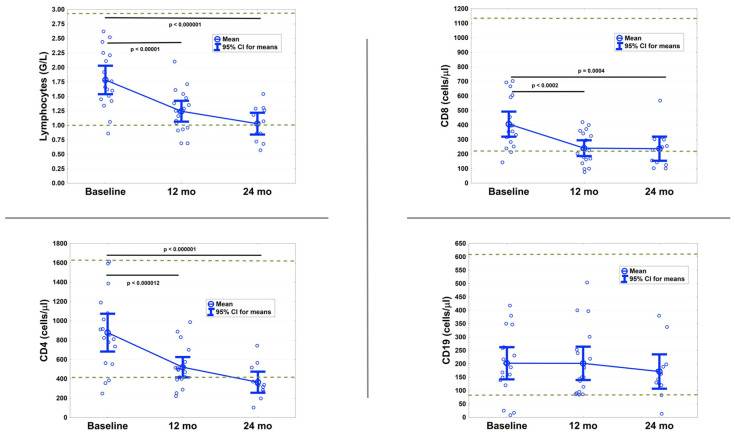
Lymphocyte subset analysis showing the impact of cladribine (CLAD) on circulating lymphocytes, cytotoxic T cells (CD8+), T helper cells (CD4+) and B cells (CD19+) before CLAD administration and at the end of each treatment year. CLAD tablets were administered at baseline and at months 1, 12 and 13 according to manufacturer’s recommendations. Dotted lines represent the normal range.

As expected, CLAD had a pronounced impact on the peripheral immune components (Figure 2). Absolute lymphocyte counts were significantly reduced (T1: *p* < 0.00001; T2: *p* < 0.000001). During the course of the 24 months follow-up, three patients developed Grade 1 (800–999/µL) and four patients a Grade 2 (500–799/µL) lymphopenia. No higher-grade lymphopenias were observed.

Expectedly, absolute numbers of CD4 + Th and CD8 + cT cells were significantly decreased at 12 months (*p* < 0.000012 and *p* < 0.0002) and 24 months (*p* < 0.000001 and *p* = 0.0004), whereas CD19+ B cell numbers were not significantly altered at T1 or T2 compared to baseline.

### 3.5. No Serious Infections Occurred

Infections were reported by six patients. Upper respiratory tract infection and herpes labialis was reported by two patients. One participant suffered from a mild COVID-19 infection and one from hand-foot-and-mouth disease. One had recurrent urinary tract infections and one patient suffered from a VZV reactivation towards the end of the second treatment year. The VZV antibody titers (cut-off > 50 mIU/mL) of this latter patient in fact increased from 710 mIU/mL at baseline and 660 mIU/mL at T1 to 10,000 mIU/mL at T2. By that time, the patient’s absolute lymphocyte count was 570 cells/µL and absolute IgG levels were relatively stable (854 mg/dL (baseline), 995 mg/dL (T1) and 892 mg/dL (T2)). This patient’s antibody levels did not affect any of the results. No serious infections occurred, and none of the participants required hospitalization.

## 4. Discussion

Despite the pronounced lymphodepleting effect [4,6], CLAD had no impact on the pre-existing humoral immunological memory in our study cohort consisting of 18 patients with MS. We assessed antibody titers to seven common pathogens before, and at 12 and 24 months after CLAD start and found no decreases over time, demonstrating a persistence of pre-treatment antibody levels. As the half-life of IgG is of approximately 3 weeks [14], our 24 months findings suggest not only that pre-existing titers are spared by CLAD, but also that the continuous production of antibodies is not affected by treatment. A retained humoral immune competence likely contributes to the capacity to cope with invasive pathogens, which is of particular importance for vaccine-preventable diseases. Our data thus provide the immunological molecular background as it is consistent with results from phase III clinical trials (CLARITY, CLARITY Extension and ORACLE-MS) and the long-term safety registry Premiere, which found a favorable safety profile and no CLAD-associated infections expect for VZV susceptibility [7,15,16,17]. Among our cohort, one patient developed herpes zoster despite specific antibody levels well beyond the specified cut-off. Moreover, this patient developed a tenfold humoral anti-VZV antibody response during infection, indicating a robustly functioning humoral system under CLAD therapy. This, on the other hand, underscores that the humoral antibody response represents only one component of immune defense, with direct cellular cytotoxicity also playing a significant role in combatting infections. In fact, treatment-induced lymphopenia after alemtuzumab administration has been associated with an increased risk of severe infections, including vaccine-preventable infections by VZV [18]. As a result, herpes prophylaxis is recommended in alemtuzumab-treated patients until lymphocyte recovery [19].

How, and to what extent, immunomodulating therapies compromise the physiological immune defense and whether they alter vaccine responses is currently an active topic of investigation. A recent study observed no CLAD-associated inhibition in antibody responses to SARS-CoV-2 immunization [20]. In the aforementioned study, the vaccine was administered 4.4 months after the last CLAD intake, a period when lymphocyte components are still affected by CLAD. Preserved antibody responses in patients receiving CLAD have now been confirmed by additional studies, after anti-SARS-CoV-2 vaccines [21] as well as after natural infections [22]. Though we did not study the course of SARS-CoV-2 antibody titers, our data suggest that once a sufficient neutralizing antibody concentration against the SARS-CoV-2 spike protein has been reached, CLAD administration does not negatively influence pre-existing titers. We conclude that the mode of action of CLAD does not come at the expense of inhibiting specific antibody production to pathogens.

Cladribine is as a purine analog, which depletes CD19+ B cells more than CD3+ T cells. Circulating B cells are rapidly reduced by over 80% before they start repopulation from week 13. T cells are reduced to a lesser extent, but they exhibit slower recovery dynamics [6]. In line with this, we found no significant B cell reductions at the end of each treatment year in the lymphocyte subset analysis, while CD4+ Th cells and CD8+ cT cells remained significantly below baseline values. These comparable lymphocyte subset data in a real-world setting make our study even more valid in terms of antibody interpretation.

In addition to retained specific antibody titers, we found no decrease in absolute IgG blood concentrations within 24 months of the first CLAD administration. In contrast to our results, reductions in serum IgG levels were associated with other high-efficacy disease-modifying MS therapies. The depletion of anti-CD20 and anti-CD52 therapies was in fact linked to antibody deficiencies, and moreover, the extent of hypogammaglobulinemia induction correlated with a higher risk of infection [23,24]. Importantly, both treatments profoundly target B cells, including pathogenic CD27+ memory phenotypes, but spare antibody producing plasma cells [3]. However, memory B cells express membrane bound immunoglobulins on their surface, which upon antigen re-encounter, can rapidly differentiate into plasma cells and induce antibody secretion [25]. While plasma cells mostly reside within supportive niches in lymphoid organs, memory B cells circulate throughout the body. A possible mechanism behind the decreased IgG synthesis associated with anti-CD20 and anti-CD52 therapies is therefore an indirect effect on plasma cell formation by depletion of their progenitor cells. However, were this the case, a similar reduction in IgG synthesis would also be expected in CLAD-treated patients, particularly owing to the improved tissue penetration of CLAD compared to monoclonal antibodies due to its small molecular size [26]. In light of these findings, we propose the following explanations: Alemtuzumab induces a more severe lymphopenia compared to CLAD [5], and peripheral B cells remain practically undetectable during anti-CD20 treatment [27]. It is therefore conceivable that the functionality of the humoral branch is only affected under certain “extreme” treatment-induced compartment changes—e.g., continuous B cell suppression during anti-CD20 therapy, or by the nearly complete depletion not only of circulating B cells but also of T lymphocytes, which contribute to shape B cell maturation and differentiation [25]—in the first weeks following alemtuzumab therapy [28].

However, we offer a note of caution. We cannot exclude that a reduced antibody synthesis associated with CLAD treatment occurs after an extended follow-up beyond 24 months from CLAD start. Other limitations are the small number of patients and dropouts over time. Additionally, the investigated cohort is relatively young, and we cannot exclude a different impact on immunological responses and memory in older patients.

## 5. Conclusions

Within the limits of the small cohort investigated, we found no impact of CLAD on pre-treatment pathogen-specific antibody titers and no effect on absolute blood IgG concentrations over 24 months, suggestive of a retained humoral memory.

## Figures and Tables

**Table 1:** Characteristics of the study population at the time of CLAD start (baseline).

No.	18
Female/Male (%)	14/4 (78/22)
Mean Age, y (±SD)	36.7 (8.8)
Mean Disease Duration, Mo (±SD)	103.6 (97.6)
Median EDSS (IQR)	1.75 (1–3.5)
Mean CD19+ B Cell Count/µL, (95% CI of the Mean); normal range: 80–616	202.2 (146.0–258.3)

CLAD = cladribine; No. = number of patients; SD = standard deviation; y = years; Mo = months; IQR = interquartile range; CI = confidence intervals.

## Data Availability

The data that support the findings of this study are available on reasonable request from the corresponding author.

## Appendix A

**Table A1:** Instruments, methods and cut-off values for pathogen-specific antibody level assessment.

Pathogen	Cute Off	Kit	Manufacturer	Instrument	Method
Measles	>150 mIU/mL	Anti-Masern ELISA (IgG)	Euroimmun *	Euroimmun Analyzer I (EUROIMMUN)	ELISA
Mumps	>1:231	Anti-Mumps ELISA (IgG)	Euroimmun *	Euroimmun Analyzer I	ELISA
VZV	>50 mIU/mL	Anti-VZV ELISA (IgG)	Euroimmun *	Euroimmun Analyzer I	ELISA
Rubella	>5 IU/mL	Alinity Rubella IgG assay	Abbott Diagnostics (Illinois, USA)	Architect i2000SR (Abbott Diagnostics)	CMIA
Hepatitis B	>100 U/L	Alinity Anti-HBs assay	Abbott Diagnostics (Illinois, USA)	Architect i2000SR (Abbott Diagnostics)	CMIA
Diphtheria	>0.01 U/mL	Anti-Diphteria Toxin ELISA (IgG)	Euroimmun *	Euroimmun Analyzer I	ELISA
Tetanus	>0.01 IU/mL	Anti-TetanusToxin ELISA (IgG)	Euroimmun *	Euroimmun Analyzer I	ELISA

VZV = varicella zoster virus; ELISA = enzyme-linked immunosorbent assay; CMIA = chemiluminescent microparticle immunoassay. * EUROIMMUN Medizinische Labordiagnostika AG, Lübeck, Germany).
